# Regional long-term analysis of dietary isotopes in Neolithic southeastern Italy: new patterns and research directions

**DOI:** 10.1038/s41598-023-34771-y

**Published:** 2023-05-16

**Authors:** M. A. Tafuri, S. Soncin, S. Panella, J. E. Thompson, I. Tiberi, P. F. Fabbri, S. Sivilli, F. Radina, S. Minozzi, I. M. Muntoni, G. Fiorentino, J. Robb

**Affiliations:** 1 Department of Environmental Biology and Mediterranean bioArchaeological Research Advances (MAReA) Centre, Sapienza University of Rome, Rome, Italy; 2 McDonald Institute for Archaeological Research, University of Cambridge, Cambridge, UK; 3 Darwin College, University of Cambridge, Cambridge, UK; 4 Polo Biblio-Museale Regionale di Lecce, Lecce, Italy; 5 Department of Cultural Heritage, University of Salento, Lecce, Italy; 6 Soprintendenza Archeologia, Belle Arti e Paesaggio per la Città Metropolitana di Bari, Bari, Italy; 7 Department of Translational Research and New Technologies in Medicine and Surgery, University of Pisa, Pisa, Italy; 8 Soprintendenza Archeologia, Belle Arti e Paesaggio per le Province di Barletta-Andria-Trani e Foggia, Foggia, Italy; 9 Department of Archaeology, University of Cambridge, Cambridge, UK

**Keywords:** Biogeochemistry, Environmental sciences, Environmental social sciences

## Abstract

Isotopic analyses of prehistoric diet have only recently reached the threshold of going beyond site-focused reports to provide regional syntheses showing larger trends. In this work we present the first regional analysis for Neolithic southeastern Italy as a whole, including both substantial original data and a review of the available published data. The results show that dietary isotopes can shed new light on a number of traditional and important questions about Neolithic foodways. First, we observe regional variations in the distribution of stable isotope values across the area, suggesting variability in the Neolithic diet. Secondly, we show that, although the plant food calorific intake was primary for these communities, animal products were also important, representing on average 40% of the total calories. Third, we note that marine fish was only minorly consumed, but that this could be an underestimation, and we observe some variability in the regions considered, suggesting differences in local human–environment interactions. People in different regions of southeastern Italy may have consumed different versions of a common Neolithic diet. Regional synthesis also allows us to take stock of gaps and new directions in the field, suggesting an agenda for Neolithic isotopic research for the 2020s.

## Introduction

Stable carbon and nitrogen isotope studies of human remains in prehistoric Italy have rapidly increased in the past two decades^1–17^. Although the isotopic evidence has greatly improved our understanding of dietary practices throughout the peninsula, this is hardly systematic, with some geographical areas and chronological phases dramatically understudied. While this calls for increased research on prehistoric Italy, we here synthesise the existing literature for one of the areas with a higher density of human and animal collagen isotope data: Neolithic southeastern Italy. This provides the chance to have a virtually uninterrupted sequence of information for a relatively limited area, which is of great archaeological interest. Indeed, in Italy, the earliest appearance of domesticated crops and animals outside their natural climatic zone is concentrated in the southeast and corresponds to increased human occupation along the southern Adriatic coast, particularly in the first half of the 6^th^ millennium BCE. There is a general consensus that Neolithic communities in Italy relied on domesticated crops and animals; but while farming appears to be central in the subsistence strategies of such communities, the role of herding and, more importantly, the contribution of marine environments is still to be clearly identified^18^.

Within a newly acquired subsistence model, could the different contribution of the same food sources to human diet help disentangle specific cultural scenarios? Further, how could local environmental phenomena participate in unravelling said complexity? Italy has a long tradition of cultural diversity. The peninsula has a varied environment, which encompasses different ecoregions, hence it is understandable that food traditions diversified. Further, besides economy and consumption, commensality may have played a role in food choices in the past: something we could associate with the concept of cuisine (*sensu* Robb^18^), by which different assortments of foods might have constituted everyday Neolithic meals, regardless of what was available on the menu.

We explore Neolithic foodways through stable carbon and nitrogen analysis of human and animal bone collagen from a large number of sites, of Early, Middle and Late phases. We do so by posing specific questions: (I) how diversified was the Neolithic diet in southeastern Italy? (II) what was the role of animal products in the diet of Neolithic communities? (III) was marine fish consumed by individuals living in proximity to the coast and was its contribution homogeneous? Part of this study stems from an earlier project, hence new data will be coupled with earlier works that were mostly focused on single case studies. Comparative analysis will be provided by published data available for prehistoric Italy.

### Carbon and nitrogen stable isotope analysis in the Mediterranean

When it comes to studying dietary habits and mobility patterns of ancient human populations, stable isotope analyses are nowadays the most deployed method, as proven by the exponential increase of articles published in the last fifty years mentioning the words ‘archaeology’ and ‘stable isotopes’^19^. Stable carbon and nitrogen isotopes in particular are capable of unravelling ecological and social questions linked to food consumption; the working principle consists in detecting the existing differences of isotopic ratios among environments, organisms and their tissues. Isotope results are reported as δ values (δ^13^C and δ^15^N) in parts per 1000 or “per mil” (‰). Thanks to how carbon fractionates depending on different photosynthetic pathways and in different ecosystems, δ^13^C (‰) values can be used to discriminate between C_3_ (~ − 28‰), C_4_ (~ − 14‰) and CAM (~ − 11‰) plants and their consumers^20^, as well as between terrestrial (~ − 7‰) and marine (~ 0‰) or freshwater (~ − 15‰) ecosystems^21^. On the other hand, δ^15^N (‰) values are mostly affected by climatic and environmental conditions and they are mainly used as a trophic level indicator^22,23^.

Once plants are eaten by animals, the carbon and nitrogen atoms composing plant tissues are used by the animals to build up their own tissues through biochemical reactions. It has been observed that both carbon and nitrogen are enriched in their heavier isotope at each step of the food web and this effect is used to assess the relative contribution of different food categories to the diet of past human individuals, also using quantitative approaches. It was initially noted that δ^13^C (‰) values of animals are enriched by + 1‰ compared to their diet^24^. However, subsequent feeding experiments and controlled field studies have shown more diversified offsets from diet to consumer tissues (Δ^13^C_tissue−diet_), ranging from + 0.5 to + 7.5‰^25–31^ depending on diet composition and on the fact that carbon is "scrambled" from all parts of the diet (i.e., carbohydrates, lipids and protein)^32^. On the contrary, the δ^15^N (‰) values of bone collagen are directly linked to protein consumption and therefore Δ^15^N_collagen−diet_ offset values are not affected by total dietary composition. As a result, feeding experiments have shown much less variable Δ^15^N_collagen−diet_ offset values compared to carbon, suggesting an overall Δ^15^N_collagen−diet_ value of + 3.4 ± 1.0‰^25,30,33–37^. However, a controlled dietary study on humans^38^ proposed a higher Δ^15^N_collagen−diet_ offset of ca. + 6‰.

On the basis of these principles, marine fish consumption can easily be detected using stable isotope analysis. However, the usual discriminants do not apply well in Mediterranean contexts. Indeed, archaeological marine fish samples from the Mediterranean have reported much lower δ^15^N (‰) values compared to those of marine fish from the Atlantic, often closer to those of terrestrial animals or manured cereals from the same area^39,40^, although the reasons behind this difference are still to be explored. Mean δ^13^C (‰) values might also be different in the Mediterranean compared to other areas of the world. It is important to note that the ancient Mediterranean coast was largely characterised by marshy lagoons (perhaps 6500 km^2^) and, in terms of food exploitation, they are twice as productive as the open sea^41^. Freshwater discharges present in coastal lagoons and wetlands determine more 13C-depleted isotope values, as observed in the fish living in their proximity^42^. Therefore, it is possible that more negative δ^13^C (‰) values of the humans from some of the coastal sites are influenced by the consumption of brackish fish with ^13^C-depleted isotope values caused by freshwater inputs. More negative δ^13^C (‰) values in the prehistoric Mediterranean were found for example in some marine fish^8,43^. The amount of fish consumed might be partially masked by the C_3_ terrestrial signal and therefore underestimated.

## Results

**Figure 1:**
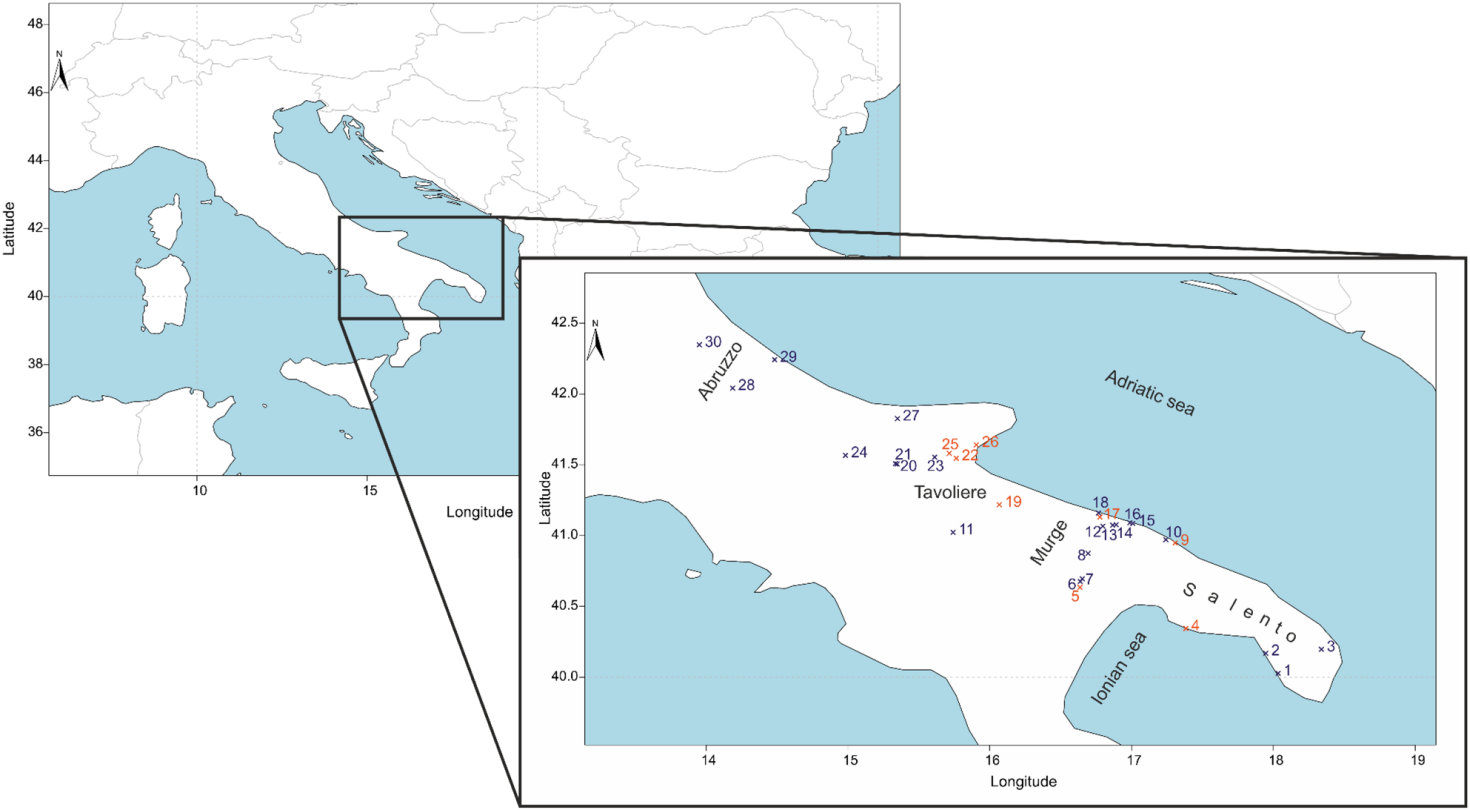
Location of the archaeological sites with human individuals analysed for stable carbon and nitrogen isotope analysis. Blue crosses indicate sites from this study, red crosses indicate sites from the literature. 1: Samari; 2: Serra Cicora; 3: Carpignano Salentino; 4: Torre Castelluccia^8^; 5: Grotta Funeraria^1^; 6: Trasano; 7: Tirlecchia; 8: Malerba-Altamura; 9: Grotta delle Mura^8^; 10: S. Barbara; 11: Diga di Rendina; 12: Balsignano; 13: Masseria Stevanato; 14: Madonna delle Grazie; 15: Cala Colombo; 16: Cala Scizzo; 17: Masseria Maselli^8^; 18: Titolo-Palese; 19: Palata^8^; 20: Foggia; 21: Ripa Tetta; 22: Fonteviva^44^; 23: Passo di Corvo; 24: Diga di Occhito; 25: Masseria Candelaro^45^; 26: Grotta Scaloria^14,45^; 27: La Torretta—Poggio Imperiale; 28: Lama dei Peligni; 29: Fossacesia; 30: Catignano. Some of the sites from this study were also previously investigated by other authors and these are: Balsignano, Ripa Tetta and Samari^8^ and Passo di Corvo^45^.

We collected human and animal bone samples from a variety of Neolithic sites from southeastern Italy (Fig. 1). Archaeological information for each site studied here is provided in the Supplementary Information S1.

This section presents the new isotopic data, which will be contextualised using existing data in the following discussion section. New data comprises 94 human samples and 16 animal samples of various wild and domesticated species (namely, *Bos* sp., *Sus* sp., *Ovis vel Capra*, *Cervus* sp. and *Capreolus* sp.) (Table S1). In the following analysis, only samples with C:N ratio between 2.9 and 3.6 are included^46^. Out of the total, this limits our results to 88 human samples and 15 animal samples, with mean δ^13^C values of − 19.6 ± 0.5‰ and δ^15^N values of 9.0 ± 1.1‰ for the humans and mean δ^13^C values of − 20.6 ± 0.7‰ and δ^15^N values of 6.4 ± 1.0‰ for the animals. Table 1 reports descriptive statistics of the data that passed the quality check presented here and of those previously published used for comparative purposes. Previously published data are reported in Table S2.

**Table 1:** Descriptive statistics of the human remains analysed in this study and those from previously published work.

Site	*n*	mean δ^13^C	median δ^13^C	1SD δ^13^C	min δ^13^C	max δ^13^C	mean δ^15^N	median δ^15^N	1 SD δ^15^N	min δ^15^N	max δ^15^N	Reference
Balsignano	2	− 19.3	− 19.3	0.1	− 19.3	− 19.2	9	9	1.2	8.1	9.8	This study; Lelli et al. 2012^8^
Cala Colombo	6	− 19.5	− 19.4	0.4	− 20.2	− 19.3	9.2	9.4	1.2	7	10.6	This study
Cala Scizzo	1	− 19.9					7.6					This study
Carpignano Salentino	1	− 19.7					9.3					This study
Catignano	3	− 20.5	− 20.1	0.9	− 21.5	− 19.9	7.3	7.5	1.5	5.7	8.7	This study
Diga di Occhito	9	− 19.9	− 20.2	0.4	− 20.4	− 19.3	9	8.8	0.7	8.4	10.8	This study
Diga di Rendina	8	− 19.7	− 19.7	0.2	− 20	− 19.4	9.4	9.3	0.4	9.1	10.2	This study
Fossacesia	1	− 19.2					9.6					This study
La Torretta–Poggio Imperiale	4	− 19.4	− 19.4	0.2	− 19.6	− 19.1	8.4	8.6	0.8	7.2	9.1	This study
Lama dei Peligni	1	− 19.1					12.3					This study
Madonna delle Grazie	4	− 19.6	− 19.5	0.4	− 20.2	− 19.2	9.5	9.6	0.7	8.8	10.1	This study
Malerba–Altamura	1	− 19.1					8.7					This study
Masseria Stevanato	2	− 20.2	− 20.2	1.3	− 21.1	− 19.2	7.3	7.3	2.8	5.3	9.3	This study
Passo di Corvo	15	− 19.1	− 19.1	0.2	− 19.5	− 18.8	8.9	8.7	0.7	7.9	10.4	This study; Tafuri et al. 2014^45^
Ripa Tetta	5	− 19.9	− 20	0.2	− 20.1	− 19.6	10.4	10	1.1	9.3	11.7	This study; Lelli et al. 2012^8^
S. Barbara	2	− 19.2	− 19.2	0.6	− 19.6	− 18.7	9	9	2.4	7.3	10.7	This study
Samari	11	− 19.1	− 19.1	0.4	− 19.8	− 18.5	9.8	9.8	0.6	8.4	10.5	This study; Lelli et al. 2012^8^
Serra Cicora	16	− 19.4	− 19.4	0.5	− 20.6	− 18.7	9.2	9.3	0.9	7.2	10.4	This study
Tirlecchia	4	− 19.6	− 19.6	0.1	− 19.7	− 19.5	9.1	9.1	0.2	8.9	9.3	This study
Titolo-Palese	11	− 19.6	− 19.5	0.4	− 20.4	− 19.1	8	8	0.7	6.3	9	This study
Trasano	7	− 19.7	− 19.8	0.2	− 20	− 19.3	9.2	9.3	0.5	8.4	9.9	This study
Fonteviva	2	− 19.4	− 19.4	0.1	− 19.4	− 19.3	10	10	0.4	9.7	10.2	Parkinson and McLaughlin 2020^44^
Grotta dell'Antenato	1	− 19.2					10.8					Arena et al. 2020^1^
Grotta delle Mura	2	− 18.7	− 18.7	1.3	− 19.6	− 17.7	7.9	7.9	0.1	7.8	8	Lelli et al. 2012^8^
Grotta Funeraria	6	− 19.5	− 19.5	0.3	− 19.9	− 19.1	9.8	9.8	0.2	9.5	10.2	Arena et al. 2020^1^
Grotta Scaloria	45	− 19.3	− 19.3	0.3	− 19.9	− 18.9	8.4	8.4	0.7	6.8	10.6	Tafuri et al. 2014^45^; Tafuri et al. 2017^16^
Masseria Candelaro	23	− 19.2	− 19.2	0.3	− 19.9	− 18.2	9.3	9.2	0.8	7.9	11.4	Tafuri et al. 2014^45^
Masseria Maselli	1	− 19.6					8.1					Lelli et al. 2012^8^
Palata	2	− 19.3	− 19.3	0.3	− 19.5	− 19.1	9.1	9.1	0.7	8.6	9.6	Lelli et al. 2012^8^
Torre Castelluccia	1	− 18.8					8.4					Lelli et al. 2012^8^

**Figure 2:**
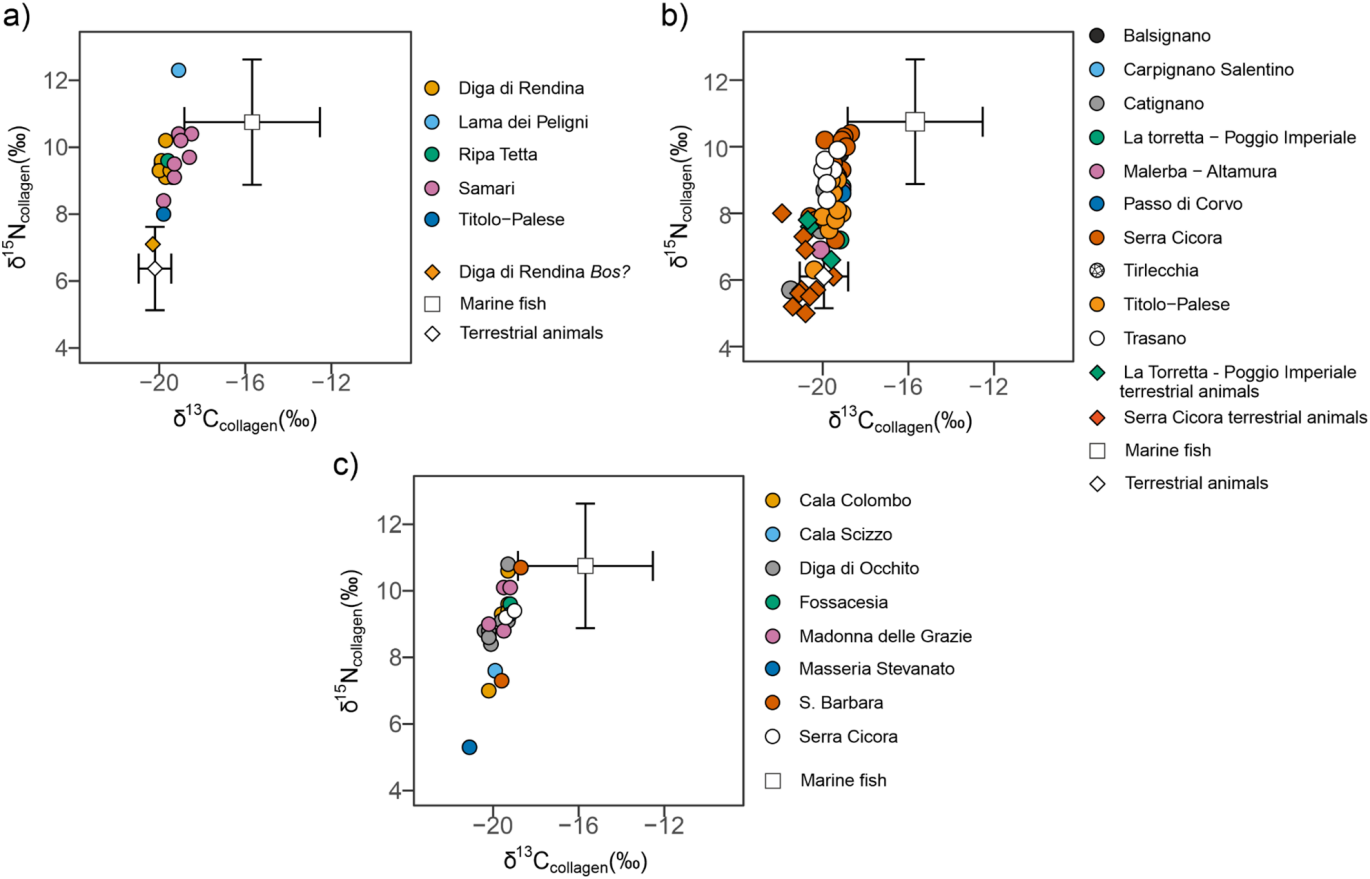
Early (**a**) Middle (**b**) and Late (**c**) Neolithic human individuals and faunal remains analysed for the present study. Early (**a**), Middle (**b**) and Late (**c**) Neolithic animal remains and marine fish from Italy from previous studies^8,45^ were included as mean values. Bars represent 1SD.

We explored the new data in relation to previously published domestic and wild fauna^8,45^, and present them below distinguishing between Early, Middle and Late Neolithic phases (Fig. 2).

### Early Neolithic (ca. 6000–5500 BCE)

In Fig. 2a are reported δ^15^N (‰) and δ^13^C (‰) values of Early Neolithic samples. 18 human individuals were successfully analysed and 1 animal sample tentatively identified as *Bos* from Diga di Rendina. The most ^13^C-depleted sample is the Early Neolithic adult female from Titolo-Palese (T9, − 19.8‰), followed by the 8 individuals from Diga di Rendina (mean δ^13^C = − 19.7 ± 0.2‰), the adult female from Ripa Tetta (IT27, − 19.6‰) and by the adult female from Lama dei Peligni (LdP, − 19.1‰). Finally, the individuals from Samari present the highest δ^13^C mean value of the Early Neolithic group (mean δ^13^C = − 19.1 ± 0.4‰). As for nitrogen, Lama dei Peligni exhibits the highest δ^15^N value (12.3‰), followed in descending order by Samari (mean δ^15^N = 9.7 ± 0.7‰), Ripa Tetta (IT27, 9.6‰), Diga di Rendina (mean δ^15^N = 9.4 ± 0.4‰) and Titolo-Palese (T9, 8‰).

### Middle Neolithic (ca. 5500–4300 BCE)

The Middle Neolithic sample is the largest dataset for human individuals (*n* = 48) (Fig. 2b). One individual from Serra Cicora (SC23) cannot be assigned to either the Middle or the Late phase of the Neolithic due to a lack of contextualisation. This individual is here included for visualisation but excluded from the following descriptive statistics. Fourteen associated animal samples were also successfully analysed, namely one probable *Bos* from Catignano, 3 (*Sus*, *Bos* and *Ovis vel Capra*) from La Torretta—Poggio Imperiale and 10, ranging between *Bos*, *Cervus*, *Sus* species and *Ovis vel Capra*, from Serra Cicora.

The highest δ^13^C values are found at Passo di Corvo (*n* = 2, mean δ^13^C = − 19.2 ± 0.1‰), followed in descending order by Balsignano (BA8, − 19.2‰), the 4 individuals from La Torretta—Poggio Imperiale (mean δ^13^C = − 19.4 ± 0.2‰), 13 of the individuals from Serra Cicora (mean δ^13^C = − 19.4 ± 0.5‰), the 10 Middle Neolithic individuals from Titolo-Palese (mean δ^13^C = − 19.6 ± 0.4‰), the 4 individuals from Tirlecchia (mean δ^13^C = − 19.6 ± 0.1‰), the two individuals from Malerba-Altamura (mean δ^13^C = − 19.6 ± 0.7‰), by the seven individuals from Trasano (mean δ^13^C = − 19.7 ± 0.2‰), by the individual from Carpignano Salentino (SC28, − 19.7‰) and finally by Catignano (*n* = 3, mean δ^13^C = − 20.5 ± 0.9‰). The lowest δ^15^N values are found at Catignano (mean δ^15^N value = 7.3 ± 1.5‰) and these are followed by Malerba-Altamura (mean δ^15^N value = 7.8 ± 1.3‰), Titolo-Palese (mean δ^15^N value = 8.0 ± 0.8‰), La Torretta-Poggio Imperiale (mean δ^15^N value = 8.4 ± 0.8‰), Passo di Corvo (mean δ^15^N value = 8.6 ± 0.0‰), Tirlecchia (mean δ^15^N value = 9.1 ± 0.2‰), by Trasano (mean δ^15^N value = 9.2 ± 0.5‰), Serra Cicora (mean δ^15^N value = 9.3 ± 1‰), by the individual from Carpignano Salentino (SC28, 9.3‰) and finally by Balsignano (BA8, 9.8‰).

### Late Neolithic (ca. 4300–3800 BCE)

From the Late Neolithic, 27 human individuals were successfully analysed (Fig. 2c). Unfortunately, no animal remains were available for this dataset and, to our knowledge, fauna dated to the Late Neolithic is also lacking in the literature. The lowest δ^13^C values are found at Masseria Stevanato (*n* = 2, mean δ^13^C = − 20.2 ± 1.3‰) followed in ascending order by Diga di Occhito (*n* = 9, mean δ^13^C = − 19.9 ± 0.4‰), the sub-adult from Cala Scizzo (BA21, − 19.9‰), Madonna delle Grazie (*n* = 4, mean δ^13^C = − 19.6 ± 0.4‰), Cala Colombo (*n* = 6, mean δ^13^C = − 19.5 ± 0.4‰), the female adult from Fossacesia (IT8, − 19.2‰), the two Late Neolithic individuals from Serra Cicora (mean δ^13^C = − 19.2 ± 0.3‰), and finally by S. Barbara (*n* = 2, mean δ^13^C = − 19.2 ± 0.6‰). The highest δ^15^N values exhibited by Fossacesia (9.6‰) followed in descending order by Madonna delle Grazie (mean δ^15^N = 9.5 ± 0.7), Serra Cicora (mean δ^15^N = 9.3 ± 0.1), Cala Colombo (mean δ^15^N = 9.2 ± 1.2‰), S. Barbara (mean δ^15^N = 9.0 ± 2.4‰), Diga di Occhito (mean δ^15^N = 9.0 ± 0.7‰), Madonna delle Grazie (mean δ^15^N = 8.8 ± 1.6‰), Cala Scizzo (7.6‰) and finally by Masseria Stevanato (mean δ^15^N = 7.3 ± 2.8‰).

## Discussion

### *Cucina tipica* in the Neolithic?

Even when people in a region share a common tradition for producing foods, and a common repertory of ingredients and techniques for preparing them, there can be strong local differences in how they eat. For example, recent historic cuisine in peninsular Italy is based on a limited, common range of resources (grains, vegetables, animal products such as cheese, pork and beef) with some regional variations (notably, the availability of fish, and the use of different grains suitable for different local climates). In spite of this, there are strong regional traditions of *cucina tipica*, or local cuisine, which combine and emphasise these in distinctive ways. Roman writers suggest that there were regional variations in foodways in Classical times, and as food is an important part of the habitus and the reproduction of communities, there is no reason to suppose that it was otherwise in prehistoric periods. Much of regional foodways may be invisible archaeologically, though we might perhaps see local patterns of material culture (e.g. pottery forms), animal remains (e.g. butchery patterns), or food consumption.

**Figure 3:**
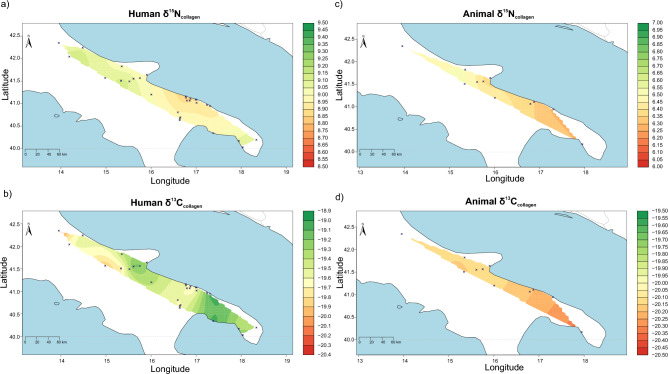
Maps with the distribution of stable nitrogen (**a**,**c**) and carbon (**b**,**d**) isotope data from Neolithic southeastern Italy, of humans (**a**,**b**) and animals (**c**,**d**), created using the app IsoMemo, tool AverageR (https://isomemoapp.com/app/iso-memo-app). Data comes from this study and from Lelli et al.^8^, Tafuri et al.^45^, Arena et al.^1^ and Parkinson and McLaughlin^44^.

The only area of Italy where the isotopic data for the Neolithic are dense enough to examine is southeastern Italy, including Puglia and the adjacent regions of Basilicata and Abruzzo. To evaluate regional differences, we explored the variability of δ^13^C (‰) and δ^15^N (‰) of humans throughout the southeastern area of the Peninsula, coupling data reported here with those of published works. The data were elaborated using the Bayesian model AverageR (see Materials and Methods for a description^47,48^, and the outputs presented in Fig. 3a,b. Terrestrial animals δ^13^C (‰) and δ^15^N (‰) values were also explored as these most likely reflect the local environment and therefore can help in the discussion of the human data. Models for the animals are reported in Fig. 3c,d. Both δ^15^N (‰) and δ^13^C (‰) values in animals do not differ according to study area (Fig. 3c,d). This could suggest that the difference in climatic and environmental conditions from one area to another is not so marked to be reflected in the isotopic values of animals, also according to modern climatic data^49^. However, the animal assemblage is limited considering the area of investigation, therefore the evidence is not strong enough to exclude climatic or environmental variations at this stage. On the contrary, the human δ^15^N (‰) and δ^13^C (‰) values appear to vary slightly but significantly from one region to another. The differences visible from the maps were also explored using descriptive statistics (Table S3), plots with mean values and associated 95% CIs (Fig. S1) and pairwise comparisons using Wilcoxon rank sum test (Table S4)^50^. Inland Murge and coastal Tavoliere differ by their mean values, as well as Salento from the coastal Murge and from the coastal Tavoliere (Fig. S1, Tables S3 and S4, p-values < 0.05). Higher δ^15^N (‰) values in the inland Murge and in the inland Tavoliere could suggest a slightly higher consumption of animal products in these regions; in fact, a stronger reliance on animals in the uplands of the Murge has been already discussed^51^. The δ^15^N (‰) values from Salento are higher than those from the coastal areas of the Murge and the Tavoliere. This could suggest higher consumption of marine products compared to other sites, again close to the sea, but from a different area. As for the distribution of δ^13^C (‰) values, differences are found between the coastal Murge and the coastal Tavoliere, as well as between the coastal Tavoliere and the inland Murge and between the inland Murge and the Salento area (Fig. S1, Tables S3 and S4, p-values < 0.05). The coastal Tavoliere and the Salento areas have higher mean δ^13^C (‰) values, which is not surprisingly observed for the coastal Murge (Fig. 3b). Again, this suggests that although these Neolithic communities had equal access to marine resources, this did not translate into similar dietary habits.

As expected, more negative values are found inland, supporting the lack of marine fish consumption. An explanation for the differences in δ^13^C (‰) values in the three different coastal areas, namely the Tavoliere, the Murgian coast and Salento could be found in the natural environment. The area of the Tavoliere and of Salento are characterised by extensive wetlands and coastal lagoons^52–54^. As already mentioned, wetlands are more productive than the open sea^41^ and they are also more easily accessible, therefore representing an important potential source of subsistence for those living in these landscapes. On the contrary, the Bari coast (coastal Murge) comprised a more limited marsh area^52^, which probably resulted in the open sea being the only viable option for the procurement of aquatic resources. Curiously, the coastal area of the Tavoliere which reports among the highest δ^13^C (‰) values among the areas explored (Fig. 3b), where extensive marshes are present, is also one of those that show the lowest δ^15^N (‰) values (Fig. 3a). This seems to suggest that the fish consumed by these local communities is most likely composed of low trophic level organisms, such as benthic bivalves, which are abundant in estuaries and lagoons^55–57^.

The isotopic evidence suggests small variation of a common Neolithic diet. One possible reason might include proximity to the sea, estuaries and lagoons. Another might include the social role of food in different kinds of communities, for instance among the large groups of the ditched villages of the Tavoliere and of the inland Murge that may have held large social gatherings involving meat consumption (e.g. feasts) more often than more dispersed, smaller communities did. This is a preliminary hypothesis; we would suggest  validating it by further investigation, including new isotope data from animals that could confirm that the differences we are observing are not related to environmental and climatic factors, and by integration with archaeological evidence of other kinds (e.g. pottery repertories; animal bones including fish and shellfish; botanical remains; food cooking facilities on sites; lipid analysis).

### The role of animal products in the diet

One of the main issues in the discussion of the Neolithic diet is the role of animals in the food economy. Neolithic people in Italy, and throughout much of Europe, had a suite of domesticated animals, including cattle, pigs, sheep, goats, and dogs (though dogs seem to have been eaten rarely). They also had access to wild game, including deer, wild pig, and small animals. But how much did these contribute to their diet? Did they eat large quantities of animal products, or were they mostly subsisting on grains and vegetables, with meat reserved mostly for social occasions? Moreover, recent studies are suggesting that secondary animal products were important in the Neolithic Mediterranean basin^58,59^.

**Figure 4:**
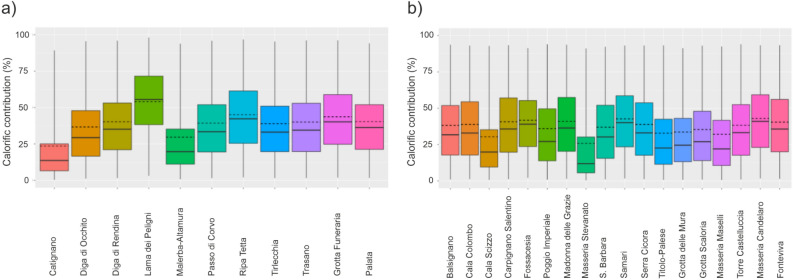
Calorific contribution (%) of terrestrial animal products in Neolithic individuals from southeastern Italy divided by sites, inland (**a**) and coastal (**b**). Boxes represent a 68% credible interval (corresponding to the 16th and 84th percentiles) while the whiskers represent a 95% credible interval (corresponding to the 2.5th and 97.5th percentiles). The horizontal continuous line represents the median (50th percentile) while the horizontal discontinuous line represents the mean.

To estimate in calorific terms the contribution of animal products to the diet of Neolithic southeastern Italian groups, we applied a Bayesian mixing model to the isotopic data (see Materials and Methods for the description of the model). It was necessary to exclude marine fish contribution from the estimations on inland sites since there is no evidence of fish consumption from these sites (i.e., no access to marine resources), therefore results are here divided in two groups, reported in Fig. 4a,b for inland and coastal sites, respectively. On average, animal products contributed to around 40% of the total calories in the diet of Neolithic individuals from inland and coastal sites (Table S5 and Fig. 4). The results suggest that animal products were not only available but also easily accessible during the Neolithic in southeastern Italy. Therefore, cereals, although contributing significantly, were not the only source of energy for these early farmers, as our models show. Our estimates do not differ from the conclusions of other studies that explore the Neolithic diet in other parts of Europe. For example, using stable isotope analysis of human bone collagen, it was observed that the majority of the diet of Neolithic communities in England and Scotland have an animal origin^60–62^ and considerable consumption of animal proteins was suggested in Neolithic Switzerland^63^, Germany^64–66^, Hungary^67^, north and central France^68–70^, Spain^71,72^, Greece^73^ and Croatia^74^. Recently, animal products have been estimated to contribute more than 50% of the diet in Neolithic Malta, probably due to a high reliance on dairy^75^.

Unfortunately stable isotopes of bone collagen cannot be used to discriminate between different animal products (in this case, meat and dairy). To answer this question other types of analyses and other kinds of material need to be studied, for example the carbon isotopes of lipids from ceramics, and age at death profiles of animal remains at each site. Differences can be outlined between sites in some cases. For example, the diet of a female from Lama dei Peligni, one of the earliest Neolithic individuals recovered in Italy, is composed of ~ 55% of calories of animal origin, probably still influenced by the earlier Mesolithic tradition (Fig. 4a). This signifies that the typical ‘Neolithic diet’ was not spread uniformly in the area and that cultural and/or social variables might have influenced these communities, as also suggested by the regional analysis. We should acknowledge however the large uncertainties associated with these estimates since the two sources used in the model, C_3_ cereals and C_3_-feeding animals, show very similar isotopic values (Table S5 and Fig. 4). Analyses at a higher resolution, such as Compound Specific Stable Isotope Analysis of Amino Acids (CSIA-AA) are needed in order to confirm and better define this scenario^76^. We should also remember that by analysing δ^13^C (‰) and δ^15^N (‰) values we are mainly exploring the protein component of diet^77^. This limits our understanding of plant consumption (notably cereals and legumes) where proteins are low, but they represent the staple for Neolithic people. To explore plant consumption, different types of analysis are needed, for example carbon isotope analysis of apatite^78–80^.

### Use of marine resources?

**Figure 5:**
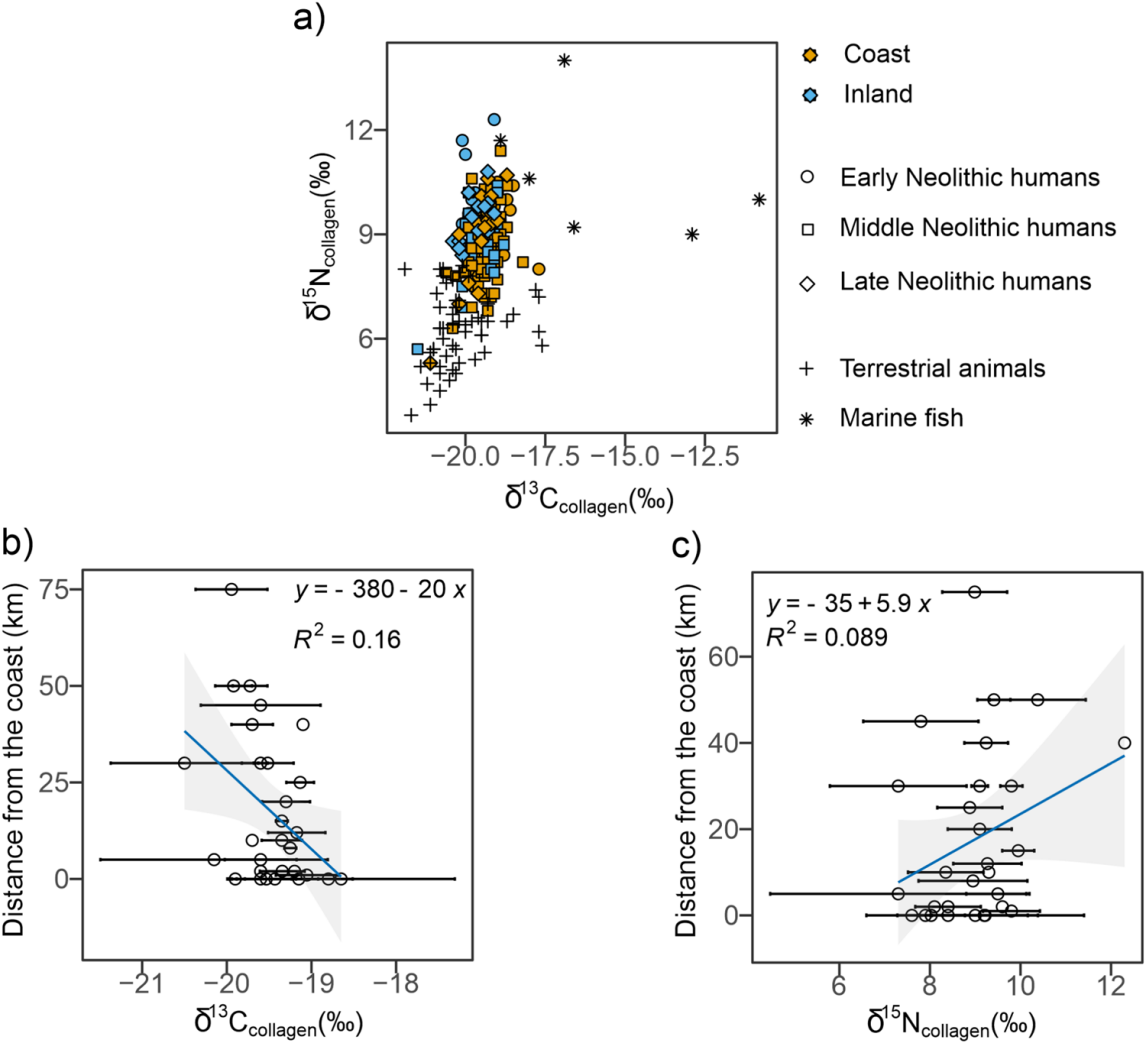
Scatterplot of δ^13^C (‰) and δ^15^N (‰) values of Early (circle), Middle (square) and Late (diamond) individuals also differentiating between coastal (orange) and inland (light blue) sites; terrestrial animals from this study and the literature^8,45^ are also plotted (crosses) as well as marine fish from the Adriatic^8^ (**a**). Correlation between δ^13^C (‰) and δ^15^N (‰) and distance to the coast (km) is explored in (**b**) and (**c**), respectively. The grey bands represent the 95% confidence interval bands. Data plotted comes from this study and from^1,8,44,45^.

A major puzzle for prehistoric food use is whether Neolithic people were using marine sources. An increasing number of isotopic studies from around Europe show that, even when they lived coastally in rich marine environments, Neolithic people lived principally on terrestrial resources^62,74,81–85^. This was a major change from Mesolithic lifeways: overall, isotopic studies have demonstrated that a gradual decline in the exploitation of marine resources at the transition to the Neolithic is observed for the Mediterranean^82,86–88^, with fishing becoming more sporadic and mostly opportunistic^89^. It cannot be ignored that marine protein consumption is believed to be limited in the Mediterranean already in the Mesolithic compared to their Atlantic and Baltic counterparts^9,10,86,90^. However, a recent study using a higher resolution approach (CSIA-AA) has shown that aquatic resources were indeed exploited^91^. This gradual decline at the transition to the Neolithic may be ascribed to cultural practices or organisational factors^83,85,92^. Food procurement from an open sea environment clearly entailed greater risk and might have resulted in limited exploitation. As discussed by Galili et al.^93^, it might be that “fishing was a low preference mode of production, to which Neolithic communities turned only once the quantity and/or quality of terrestrial resources were reduced or impaired”. To explore the question in Neolithic southeastern Italy, we plotted all the data according to proximity to the coast, since both δ^13^C (‰) and δ^15^N (‰) would increase values according to the accessibility of marine resources (Fig. 5). What we observe is no substantial differences when we explore data in a chronological perspective (Fig. 5a) and a lack of correlation between both δ^13^C (‰) and δ^15^N (‰) and distance to the coast (km) (Fig. 5b,c, R^2^ = 0.16 and *R*^2^ = 0.089, respectively) indicating that marine environments were not exploited substantially and equally by all the communities living in their proximity. The Bayesian mixing model suggests an overall low marine contribution, with some nuance at an inter-site level (Fig. S2 and Table S5). We should consider however that the marine fish consumed in those sites characterised by a lagoon environment could present more ^13^C-depleted δ^13^C (‰) values compared to the marine fish specimens used in the model (which were the only available for the area of investigation and with a different chronology), and therefore the marine contribution is possibly underestimated. In general, what emerges is a certain degree of variability in the consumption of marine resources at coastal sites from Neolithic southeastern Italy.

## Conclusion

This paper has shown the distinctive value of the analysis of a large isotopic dataset, combining new and previously published results, to evaluate regional patterns of food consumption. First, we observed regional variations throughout southeastern Neolithic Italy that are most likely not directly caused by climatic or environmental conditions but rather by how the humans adapted according to the local environment and climate. This suggests the possibility of regional variations in the Neolithic diet, even within a relatively small region with a homogeneous cultural background^94^. Second, Bayesian statistics was used to evaluate the contribution of animal products in the diet of Neolithic people from the area. The results suggest a certain degree of variability across the assemblage and that animal products were certainly not of minor importance in the diet (40% on average of the total calories have an animal origin), implying that cereals and legumes were not the only source of energy for Neolithic communities from southeastern Italy. Finally, we explored marine fish consumption in the area. Our dataset suggests that marine resources were only minorly exploited but that this is likely an underestimation. Some variability in marine fish consumption was observed, and this might be related to differences in the environment (the presence of lagoons makes it easier to access fish) or cultural preferences.

These results are preliminary and need to be reinforced by new material (plant and fish remains in particular, but also more terrestrial animals) and by higher resolution analyses, such as CSIA-AA, which has the potential to better define the differences observed, discriminate and quantify the contribution of terrestrial animals, plants and marine fish in the diet of Neolithic communities^76^. Nonetheless, a general picture of ‘local’ traditions within a larger model appears to be confirmed^8,45^. People adopted diversified cuisines, according to a shared habitus, that might have been specific to a network of sites in small areas (e.g., the Murge region or the Tavoliere and Salento). The heterogeneity of dietary habits between communities living in similar environments suggests that socio-cultural factors might have played a key role in food choices and/or farming and herding practices, regardless of local availability.

## Materials and methods

### Collagen extraction

For the majority of the samples, collagen extraction for isotopic analysis followed the Longin's^95^ method. In brief, cortical bone (ca. 0.5 g) was cleaned by sand abrasion and demineralized in 0.5 M aq. HCl at 4 °C for several days. The samples were then rinsed to neutral pH. Due to the poor preservation of the human remains from Titolo-Palese, ethylenediaminetetraacetic acid (EDTA) was used instead of HCl following Tuross^96^. These samples were decalcified in 50 mL 0.5 M EDTA at pH 7.4 for one to four weeks, changing the solution every 4 days. Once decalcified, samples were washed with deionised water 14 times, and left in water overnight. All samples were gelatinized in pH 3 water at 75 °C for 48 h. The collagen solution was filtered off with 5–8 μm Ezee filters, then frozen, and freeze dried. Stable carbon and nitrogen isotope ratios were measured using an automated elemental analyser coupled in continuous-flow mode to an isotope-ratio-monitoring mass-spectrometer. The majority of the analyses were carried out at the Godwin Laboratory, University of Cambridge. Instrument used was a Costech Elemental Analyzer coupled to a Thermo Finnigan MAT253 Mass Spectrometer. Triplicate reproducibility is less than 0.2‰ for both isotopes. The isotopic standards used are: International Atomic Energy Agency (IAEA) standards of caffeine and glutamic acid for carbon and nitrogen; in-house laboratory standards of nylon, alanine, and bovine liver standard for carbon, nitrogen, and atomic C:N ratios. The samples from Titolo-Palese were analysed at SUERC. Here, stable carbon and nitrogen isotopic compositions were determined on a Delta V Advantage continuous-flow isotope ratio mass spectrometer coupled via a ConfloIV to an IsoLink elemental analyser (Thermo Scientific, Bremen) following Sayle et al.^97^. The International Atomic Energy Agency (IAEA) reference materials USGS40 (L-glutamic acid, δ^13^C_V-PDB_ = − 26.4 ± 0.0‰, δ^15^N_AIR_ = − 4.5 ± 0.1‰) and USGS41a (L-glutamic acid, δ^13^C_V-PDB_ = 36.6 ± 0.1‰, δ^15^N_AIR_ = 47.6 ± 0.2‰) were used to normalise δ^13^C and δ^15^N values. Normalisation was checked using the marine collagen USGS88 (δ^13^C_V-PDB_ = − 16.1 ± 0.1‰ and δ^15^N_AIR_ = 15.0 ± 0.1‰) and the porcine collagen USGS89 (δ^13^C_V-PDB_ = − 18.1 ± 0.1‰ and δ^15^N_AIR_ = 6.3 ± 0.1‰), which gave the values: USGS88, δ^13^C_V-PDB_ = − 16.3 ± 0.1‰ and δ^15^N_AIR_ = 15.2 ± 0.1‰ and USGS89, δ^13^C_V-PDB_ = − 18.2 ± 0.1‰ and δ^15^N_AIR_ = 6.4 ± 0.2‰. Stable isotope concentrations are measured as the ratio of the heavier isotope to the lighter isotope relative to an internationally defined scale, Vienna Pee Dee Belemnite (VPDB) for carbon and Ambient Inhalable Reservoir (AIR) for nitrogen (Hoefs & Hoefs, 1997). Isotope results are reported as δ values (δ^13^C and δ^15^N) in parts per 1000 or “per mil” (‰). Results are reported in Table S1. Previous isotopic measurements on Neolithic humans and animals are reported in Table S2.

### Chronological and geographical assessment

Humans and animals were classified as ‘Early’ ‘Middle’ or ‘Late’ Neolithic following radiocarbon dates when available, and material culture when they were not (Table S1)^98^. The limited availability of radiocarbon dates for many prehistoric Italian sites is in part a result of well-developed material culture typologies; combined, they provide a good estimation of phasing and allow us to explore dietary variability throughout time. We also provide a new radiocarbon date of one sample of human bone (OCH2) from Diga di Occhito, dated to 5060 ± 40 BP (Beta-288147: 3962–3715 cal BC, 95.4%), placing the site within the Late Neolithic^99^ (Fig. S3).

Coordinates of the sites were provided by the archaeologists or derived from previous publications.

Where these were not available we used the coordinates of the closest town/village or landmark from the site.

### Plotting and statistical analysis

Scatter plots, descriptive statistics and statistical tests were carried out using *RStudio*, *R* version 4.0.3 using the packages *dplyr*, *tidyr, Hmisc,* and *ggplot2*.

### Regional analysis and mixing models

To answer the archaeological questions, we used two different tools from the open access application *IsoMemo* (https://isomemoapp.com/app/iso-memo-app). *AverageR* was used to explore regional variability of δ^13^C (‰) and δ^15^N (‰) values as previously described^47,48^. We used the default options with the exception of the *smooth type* (planar), *extrapolation behaviour* (constant) and we used the Bayesian model with the number of MCMC iterations set to 5000 for the analysis of the data. *ReSources* was used instead to quantitatively evaluate the diet of Neolithic individuals using a routed and concentration-dependent model^100,101^. We used terrestrial animal products, C_3_ cereals and marine fish as possible food sources for those individuals from archaeological sites located in proximity to the coast (km from the coast ≤ 15) while we only relied on terrestrial animal products and C_3_ cereals for those considered inland sites (km from the coast > 15). δ^13^C (‰) and δ^15^N (‰) values of the animals from this and previous studies were used to represent the “animal products” category after having checked that the values were uniform across southeastern Italy using regional analysis. As for the ‘C_3_ cereals’ source, since these are dramatically lacking from the area of investigation, we relied on published wheat and barley δ^13^C (‰) and δ^15^N (‰) values from a Greek Neolithic context^102^. The analysis of carbon and nitrogen stable isotopes of local plant remains is essential^103^, and here we urge for this. δ^13^C (‰) and δ^15^N (‰) values of local marine fish specimens from Lelli et al.^8^ were used to represent the ‘marine fish’ category. Unfortunately, the marine fish used belongs to an older chronology but it represents the only opportunity to use a local baseline for this food category. The sources δ^13^C (‰) and δ^15^N (‰) values were corrected to represent the actual values in the tissues consumed using the recently updated offsets from Soncin et al.^76^. Uncertainties associated with the source values are 1SD. The offset Δ^15^N_collagen-diet_ was set at + 5.5 ± 0.5‰, with 100% contribution from protein, and the offset Δ^13^C_collagen-diet_ was set to + 4.8 ± 0.5‰, with 74 ± 4% contribution from protein and 26 ± 4% from lipids and carbohydrates^104^. Concentrations expressed as dry weight (%) are the same of those reported in Soncin et al.^76^ using the USDA National Nutrient Database for Standard Reference (available at https://fdc.nal.usda.gov/) with uncertainties being the standard error. As for model parameters, we used the default options with the exception of the following: model type: Individual targets (no shared info); Source contribution distribution: deselected “Optimal objective prior”; Covariates model: fixed intercept (cat. vars), fixed slope (num. vars).

## Supplementary Information

Supplementary Information.

Supplementary Information 1.

Supplementary Information 2.

Supplementary Information 3.

Supplementary Information 4.

Supplementary Information 5.

Supplementary Information 6.

Supplementary Information 7.

Supplementary Information 8.

Supplementary Information 9.

Supplementary Information 10.

## Data Availability

The authors confirm that the data supporting the findings of this study are available within the article and/or its supplementary materials.
