# Supplementary material for: Regional long-term analysis of dietary isotopes in Neolithic southeastern Italy: new patterns and research directions
Source: Sci Rep. 2023 May 16;13:7914. doi: 10.1038/s41598-023-34771-y (PMC10188610; doi:10.1038/s41598-023-34771-y)
Supplement: Supplementary file 1 — Supplementary Information. [file 41598_2023_34771_MOESM1_ESM.docx]

Archaeological and anthropological information

In the following, we provide information on the individuals that were sampled for carbon and nitrogen stable isotope analysis and on the sites they came from, differentiating according to the Early, Middle and Late Neolithic phases which were employed for data analysis.

#### **Early Neolithic sites**

**Diga di Rendina**: The village of Rendina is located in the Materano area, on the left bank of the Ofanto river, province of Potenza. 8 human individuals and 1 animal bone were analysed for stable isotopes. REN1 (archaeological ID: dep. 1 F) is an adult female; REN2 (archaeological ID: dep. 1 M) is an adult male; REN3 (archaeological ID: dep. 3 juv.) is a 3-6 year old child; REN4 (archaeological ID (dep. 2 juv.) is a 10-14 year old juvenile; REN5 (archaeological ID: A1 dep. 3 skull) is an adult of undetermined sex; REN6 (archaeological ID: skull from N12) is undetermined; REN7 (archaeological ID: A1 dep. 3 sito 3 area A) is an adult of undetermined sex, and REN8 (archaeological ID: ZR73 sepoltura 2) is also of undetermined sex. All the samples provided good quality collagen and therefore were all included in the data analysis.

**Favella della Corte**: This individual was found in the Early Neolithic village of Favella della Corte, province of Cosenza, and it represents one of the oldest burials found in Italy to this date (6th millennium BC) [(Tinè and Tinè, 1991)](https://paperpile.com/c/QAa3wz/gkbOu). The individual (FdC) was identified as an adult male [(Scarsini *et al.*, 2008)](https://paperpile.com/c/QAa3wz/pfGZ). The C/N ratio suggests good quality collagen and therefore the individual was used for the data analysis.

**Lama dei Peligni:** Collagen was successfully extracted from the individual known by the name of “Fonterossi 1” or “Uomo della Maiella” (LdP). The skeleton was found *ca.* 1.5 m below the Neolithic village of Fonterossi (Chieti) and it was dated 6540 ± 80 BP [(Manzi and Macchiarelli, 1986)](https://paperpile.com/c/QAa3wz/lcxXZ) and was attributed to an adult female [(Manzi and Macchiarelli, 1986; Bruner and Manzi, 2003)](https://paperpile.com/c/QAa3wz/lry5m+lcxXZ). The C/N ratio suggests good quality collagen and therefore the individual was used for the data analysis.

**Ripa Tetta:**. Ripa Tetta is a ditched village located on a terrace by the Vulgano torrent in the Apulian Tavoliere, province of Foggia [(Tozzi, 2015)](https://paperpile.com/c/QAa3wz/Rdth). Collagen was extracted from four individuals and one *Bos* sample but only collagen from individual Z (IT27), an adult female, was considered good enough to be included in the analysis.

**Samari**: Samari is located in Contrada Samari, Gallipoli, province of Lecce [(Orlando, 2002)](https://paperpile.com/c/QAa3wz/nVzA).

9 individuals from the site were selected for isotope analysis. These include seven adult males (IT33, archaeological ID: EE; IT34, archaeological ID: FF; IT35a, archaeological ID: Samari H1 14/15 GG; IT36, archaeological ID: II; IT37c, archaeological ID: NN; IT37d, archaeological ID: MM), one adult female (IT38, archaeological ID: OO) and one subadult (IT39, archaeological ID: PP). Individual FF (IT34) was excluded from the analysis due to a slightly lower collagen C/N ratio.

#### **Middle Neolithic sites**

**Balsignano**: The archaeological site of Balsignano in the municipality of Modugno (Bari), spans from the early to middle Neolithic. The individual analysed (BA8, archaeological ID: T2) is a male with an estimated age at death of 31-43 years [(Scattarella *et al.*, 2002)](https://paperpile.com/c/QAa3wz/tGASd), dated to the second half of the 5th millennium BC (KIA-13414: 5783± 39 BP and KIA- 5508± 50 BP)[(Radina, 2002, 2006; Fiorentino *et al.*, 2013)](https://paperpile.com/c/QAa3wz/vO5x+yVwL+Sbii). The C/N ratio suggests good quality collagen and therefore this individual was included in the data analysis.

**Catignano**: Catignano is a village dated to the earlier phases of the painted wares [(Pitti and Tozzi, 1976)](https://paperpile.com/c/QAa3wz/Z4t3w). Here we analysed three individuals, all used in the data analysis. Individual IT15 (archaeological ID: struttura 9A/Catignano I), is an adult female found with painted pottery. This individual was found with lesions and two trepanations on the cranium, although it probably survived the traumatic event/s for at least a year. Individual IT 16 (archaeological ID: ind. A/Catignano 2) is a child of 3-5 years old and IT 18 (archaeological ID: juv. B/Catignano 3) is a younger child of 1.5-2.5 years old [(Robb and Mallegni, 1994)](https://paperpile.com/c/QAa3wz/pvfZh). The remains of one animal identified tentatively identified as *Bos* were also successfully analysed.

**La Torretta - Poggio Imperiale**: Eight burials were recovered from the Middle Neolithic ditched village of La Torretta at Poggio Imperiale. The site is located on a small elevation overlooking the Lesina Lake, Gargano [(Tunzi Sisto and Sanseverino, 2007)](https://paperpile.com/c/QAa3wz/prTc). We analysed four humans (archaeological IDs: t.1, t.2, t.4 and t.6) and three animal remains (belonging to the species *Bos, Sus and Ovis vel Capra*). The C/N ratios suggest good collagen preservation in all the samples.

**Malerba**: The individuals analysed were recovered from the second burial from the Neolithic village of Malerba II in the municipality of Altamura, province of Bari. Both are female individuals; one is a mature adult (BA19, archaeological ID: T2A), the other is a mature-old adult (BA20, archaeological ID T2B). The burial was an oval pit grave cut into the tufa bedrock [(Sublimi Saponetti *et al.*, 2001)](https://paperpile.com/c/QAa3wz/jq6TJ). In both cases the collagen C/N ratios are indicative of good quality collagen and therefore these individuals were both included in the data analysis.

**Passo di Corvo**: Passo di Corvo is a Neolithic village on the Apulian Tavoliere, located a few kilometres from the city and province of Foggia. Two new individuals were sampled in addition to those already analysed and presented in [(Tafuri *et al.*, 2014)](https://paperpile.com/c/QAa3wz/SYZ0). These are IT6 (archaeological ID: ind. D), an adult individual of undetermined sex, and IT7 (archaeological ID: ind. E), an adult male. Both were included in the data analysis.

**Serra Cicora:** Serra Cicora is a middle Neolithic site excavated in the municipality of Nardò, province of Lecce. 16 individuals from the site were analysed and all included in the analysis based on the collagen C/N ratio. These are 10 adult females (SC1, archaeological ID: T10A; SC2, archaeological ID: T6A; SC3, archaeological ID: T8A; SC6, archaeological ID: T3B; SC7, archaeological ID: T6C; SC8, archaeological ID: T4A; SC9, archaeological ID: T3A; SC10, archaeological ID: T5B; SC11, archaeological ID: T5A; SC12, archaeological ID: T6C), one adult male (SC16, archaeological ID: T9A), and one subadult (SC4, archaeological ID: T2A). 3 individuals were analysed from burial 10 (SC14, archaeological ID: T10C 234 (25), an adult male; SC17, archaeological ID: T10B 107; SC18, archaeological ID: T10D 295, an adult male) and one individual from burial 1 (SC23, archaeological ID: T1 (280-454))[(Fabbri and Lonoce, 2013)](https://paperpile.com/c/QAa3wz/cRnX). 10 bone samples of various animals were also analysed and included in the data analysis.

**Tirlecchia**: The village was identified close to Masseria Tirlecchia. The excavations carried out at the end of the 1970s concluded that the village was occupied from the Early to the Late Neolithic [(Bernabo Brea *et al.*, 1984)](https://paperpile.com/c/QAa3wz/IwlQU). The four individuals analysed here originated from the Middle Neolithic levels and these are IT9 (archaeological ID ind. G), IT10 (archaeological ID ind. H), IT11 (archaeological ID ind. I) and IT12 (archaeological ID ind. L). The individuals are all adults, IT9 is a male, IT 10 and IT11 are of undetermined sex and IT12 is a female. All the individuals were used in the data analysis.

**Titolo-Palese**: The Neolithic village at Titolo (Palese) in the municipality of Bari, probably extended over an area of *ca.* 4 ha in total, and is today 150-200 m from the coastline. The site was in use for alternating phases between the 6th and 5th millennia BC. Eleven individuals were analysed using EDTA instead of HCl for the demineralisation step [(Tuross, 2012)](https://paperpile.com/c/QAa3wz/W7Iw). These are: T1 (archaeological ID: T.1), an adult individual of undetermined sex; T2 (archaeological ID: T.2), an adult male with an estimated age at death of 30-35 years; T3 (archaeological ID T.3), an adult male estimated to be 40-50 years old at the time of death; T4 (archaeological ID: T.4), of undetermined sex; T5 (archaeological ID: T.5), also of undetermined sex; T6 (archaeological ID: T.6), estimated to be a 40-45 year old adult male; T7 (archaeological ID: T.7), a child with estimated age at death of 6 ± 2 years; T8 (archaeological ID: T.8), an estimated 30-40 year-old female; T9 (archaeological ID: T.9), an adult female with estimated age at death of 30-35 years; T10A (archaeological ID: T.10A), an adult female estimated at 45-55 year-old at the time of death, and T10B (archaeological ID: T.10B), a child with estimated age at death of 8 ± 2 years. They were all found with good collagen preservation. T2, T6 and T9 were radiocarbon dated (see Materials and Methods) and the dates suggest that T2 and T9 belong to the Early Neolithic period, while T6 dates to the Middle Neolithic [(Radina *et al.*, 2020)](https://paperpile.com/c/QAa3wz/1J39t).

**Trasano**: The Neolithic village of Trasano is located along the road that connects the cities of Matera and Laterza, province of Matera [(Guilaine and Cremonesi, 1987)](https://paperpile.com/c/QAa3wz/g8xc). We analysed seven human individuals and all were found with good collagen preservation according to the C/N ratio. IT40 (archaeological ID: ind. QQ) is an adult male, individual IT41 (archaeological ID: ind. RR) is an adult female, individual IT42 (archaeological ID: ind. SS) is another adult female, individual IT43 (archaeological ID: ind. TT) is a 6-7 year-old child, individual IT 44 (archaeological ID: silos juv.) is an estimated 7 year old child, individual IT45 (archaeological ID: ind. VV) is an estimated 12 year old child, and IT46 (archaeological ID: ind. UU) is another adult male.

#### **Late Neolithic sites**

**Cala Colombo**: Cala Colombo is a funerary and ritual hypogeum in the district of Torre a Mare (Bari), on the coast south of Bari. The site is associated with Serra d’Alto and Diana-Bellavista pottery and dated to the second half of the 5th millennium BC [(Pesce Delfino *et al.*, 1977)](https://paperpile.com/c/QAa3wz/tcpU). Six human individuals were analysed. CC1 (archaeological ID: ind.1) is an adult male; CC3 (archaeological ID: ind. 3) is an adult female; CC4 (archaeological ID: ind. 7) is an estimated 7 year old juvenile; CC9 (archaeological ID: ind. 17) is an adult male; CC10 (archaeological ID: ind. 18) is an adult male, and CC12 (archaeological id: no code) is of undetermined sex. All the individuals were used in the data analysis for their collagen C/N ratio indicative of good collagen preservation.

**Cala Scizzo**: Cala Scizzo is a small natural cave used as a ritual site located in the Torre a Mare district, on the coast south of Bari [(Geniola and Sisto, 1980)](https://paperpile.com/c/QAa3wz/KXDD). The site was in use during the 5th millennium and the beginning of the 4th millennium BC. We analysed BA21 (archaeological ID: Ind. 1 (Juv) (4)), a subadult individual. The sample reported a collagen C/N ratio in the range and therefore included in the analysis.

**Carpignano Salentino**: This individual (SC28) was found in a rare example of a “tomba a grotticella”, rock-cut tomb of the Salento area. The individual was radiocarbon dated to 5665 ± 30 BP. This funerary practice, associated with the final phases of the Diana culture, can be chronologically dated in the area of Salento to the 5^th^ millennium BC [(Ingravallo, 2009)](https://paperpile.com/c/QAa3wz/82rdm). The individual is an adult male [(Fabbri, 2009)](https://paperpile.com/c/QAa3wz/X0l3K) and was included in the data analysis.

**Diga di Occhito**: We analysed 9 commingled human individuals from a Neolithic site known by the name of “Mulino Dabbasso” or “Diga di Occhito”, today partially submerged by the artificial lake created by a dam (the dam or “diga” of Occhito). These individuals are possibly from a mass burial [(Robb, 2007)](https://paperpile.com/c/QAa3wz/2Mfed), but the site may also represent an early example of a rock-cut tomb. Disturbance of the site prior to and following excavation precludes confirmation of the feature type or burial circumstances. The chronology of the site and of the burial is uncertain but it is possible that it belongs to the Early Neolithic due to the presence of impressed ware pottery [(Tunzi, 1999)](https://paperpile.com/c/QAa3wz/zUrR). However, we carried out radiocarbon dating of one of the bones (OCH2) and reported a Late Neolithic date (5060 ± 40 BP). Therefore, for the purpose of this paper, the human individuals were considered belonging to the Late Neolithic period. All the individuals were used in the data analysis considering their collagen C/N ratio.

**Fossacesia**: the archaeological site of Fossacesia was excavated in the province of Chieti, along the left bank of the Sangro river in proximity to the highway exit ‘Pescara-Vasto’. The individual here analysed and included in the analysis for their good collagen C/N ratio (IT8) is an adult female from the Late Neolithic levels.

**Madonna delle Grazie**: Madonna delle Grazie is a settlement of the municipality of Rutigliano (Bari). The site was in use between the 6th and the 5th millennia BC. It was first excavated in the 1970s and again in the 2010s. We analysed BA14 (archaeological ID: T1 S. Lorenzo US7 - scavo nuovo), an adult female from the new excavation; BA15 (archaeological ID: T2 Rutigliano - scavo vecchio), an adult female from the first excavation; BA16 (archaeological ID: T3 - scavo vecchio) a male individual from the first excavation, and BA17 (archaeological ID: T2 (15) - scavo nuovo), an estimated 3 year old child. All the samples were included in the analysis of the data.

**Masseria Stevanato**: The site, on the outskirts of Bari, can be dated to the Late Neolithic. A bell-shaped silo was reused for ritual depositions of pottery, worked stone artefacts, and human remains (skulls and long bones), arranged on the bottom together with *Bos* horns (LTL-624A 5149± 55 BP)[(Fiorentino *et al.*, 2013; Barbieri *et al.*, 2017)](https://paperpile.com/c/QAa3wz/bsmJ+Sbii). One human bone from this assemblage was sampled and is presented here (BA6 archaeological ID: silos). Nearby was a connected human deposition (BA7, archaeological ID: Carbonara T2) probably unintentional, and datable to the Late Neolithic (Barbieri et al. 2017). Both samples were included in the data analysis.

**Santa Barbara**: Santa Barbara is located at Polignano a Mare, province of Bari, and it represents a rare example of a ditched enclosure outside of the Tavoliere and Materano areas [(Geniola and Sanseverino, 2017)](https://paperpile.com/c/QAa3wz/jicK). Two individuals were analysed: BA22 (archaeological ID: 98 Meo - ind. 1/12 sup (5)) was probably 9-12 months old [(Barbieri, Sanseverino and Sublimi Saponetti, 2017)](https://paperpile.com/c/QAa3wz/jbtJ) and was found in the cemetery area called “Meo”, associated with Diana-Bellavista pottery; BA24 (archaeological ID: La Trappola - Saggio 2 fibula (7)) is an adult female who was recovered during the 2010 campaign from the La Trappola area, a deposit inside the ditch where Serra d’Alto pottery was found [(Barbieri, Sanseverino and Sublimi Saponetti, 2017)](https://paperpile.com/c/QAa3wz/jbtJ). Both the individuals were included in the data analysis.

[Barbieri, A. *et al.* (2017) ‘Studio antropologico delle antiche comunità di agricoltori del Neolitico pugliese’, *Riunione Scientifica Preistoria e Protostoria della Puglia*, XLVII.](http://paperpile.com/b/QAa3wz/bsmJ)

[Barbieri, A., Sanseverino, R. and Sublimi Saponetti, S. (2017) ‘I reperti umani neolitici di Santa Barbara - Polignano a Mare (BA)’, in F. Radina (ed.) *Preistoria e Protostoria della Puglia*. Atti IIPP.](http://paperpile.com/b/QAa3wz/jbtJ)

[Bernabo Brea, M. *et al.* (1984) ‘L’insediamento neolitico di Tirlecchia (Matera)’, *Rivista di Scienze Preistoriche*, 39(1-2), pp. 23–84.](http://paperpile.com/b/QAa3wz/IwlQU)

[Bruner, E. and Manzi, G. (2003) ‘Towards a re-appraisal of the Early Neolithic skeleton from Lama dei Peligni (Abruzzo, Italy). Computed tomography and 3D reconstruction of the cranium’, *Rivista di Antropologia*, 81, pp. 69–78.](http://paperpile.com/b/QAa3wz/lry5m)

[Fabbri, P.F. (2009) ‘Tafonomia di una sepoltura neolitica di Carpignano Salentino (Lecce)’, in P.F. Fabbri, C. Pagliara, and T. Scarano (eds) *Prima di Carpignano. Documentazione e interpretazione di una sepoltura neolitica*. Terra.](http://paperpile.com/b/QAa3wz/X0l3K)

[Fabbri, P.F. and Lonoce, N. (2013) ‘Gli aspetti funerari: le sepolture’, in I. Tiberi (ed.) *Serra Cicora tra VI e V millennio a. C.* Istituto italiano di preistoria e protostoria.](http://paperpile.com/b/QAa3wz/cRnX)

[Fiorentino, G. *et al.* (2013) ‘Climate changes and human–environment interactions in the Apulia region of southeastern Italy during the Neolithic period’, *Holocene*, 23(9), pp. 1297–1316.](http://paperpile.com/b/QAa3wz/Sbii)

[Geniola, A. and Sanseverino, R. (2017) ‘L’insediamento neolitico di Santa Barbara a Polignano a Mare (BA)’, *L’insediamento neolitico di Santa Barbara a Polignano a Mare (BA)*, pp. 285–291.](http://paperpile.com/b/QAa3wz/jicK)

[Geniola, A. and Sisto, A.M.T. (1980) ‘Espressioni cultuali e d’arte nella Grotta di cala Scizzo presso Torre a Mare (Bari)’, *Rivista di scienze preistoriche*, (35), pp. 125–146.](http://paperpile.com/b/QAa3wz/KXDD)

[Guilaine, J. and Cremonesi, G. (1987) ‘L’habitat néolithique de Trasano (Matera, Basilicate). Pré miers resultats’, *Actas de la XXVI Riunione Científi ca a cura dell’IIPP*, 707, p. 719.](http://paperpile.com/b/QAa3wz/g8xc)

[Ingravallo, E. (2009) ‘Le società del V millennio alla luce dei rituali funerari: la tomba di Carpignano Salentino (Lecce)’, in P.F. Fabbri, C. Pagliara, and T. Scarano (eds) *Prima di Carpignano. Documentazione e interpretazione di una sepoltura neolitica*. Terra.](http://paperpile.com/b/QAa3wz/82rdm)

[Manzi, G. and Macchiarelli, R. (1986) ‘L’«uomo preneolitico» della Maiella (Lama dei Peligni, Abruzzo). II-La datazione radiometrica dello scheletro’, *Rivista di antropologia* [Preprint]. Available at:](http://paperpile.com/b/QAa3wz/lcxXZ) <https://pascal-francis.inist.fr/vibad/index.php?action=getRecordDetail&idt=6144327>[.](http://paperpile.com/b/QAa3wz/lcxXZ)

[Orlando, M.A. (2002) ‘Impresse del Sud-Est. Samari’, in Fugazzola Delpino, M.A., Pessina, A., Tinè, V. (ed.) *Le ceramiche impresse nel Neolitico antico. Italia e Mediterraneo.* Istituto Poligrafico e Zecca dello Stato, pp. 641–650.](http://paperpile.com/b/QAa3wz/nVzA)

[Pesce Delfino, V. *et al.* (1977) ‘Antropologia della comunità neolitica di Cala Colombo’, in A. De Lucia (ed.) *La comunità neolitica di Cala Colombo presso Torre a Mare (Bari)*. Società per lo Studio di Storia Patria per la Puglia (Documenti e monografie ), pp. 96–178.](http://paperpile.com/b/QAa3wz/tcpU)

[Pitti, C. and Tozzi, C. (1976) ‘Gli scavi nel villaggio neolitico di Catignano (Pescara): nota preliminare’, *Rivista di scienze preistoriche*, (31), pp. 87–107.](http://paperpile.com/b/QAa3wz/Z4t3w)

[Radina, F. (2002) ‘L’insediamento di Balsignano’, *La preistoria della Puglia. Paesaggi, uomini e tradizioni di*, 8000, pp. 143–158.](http://paperpile.com/b/QAa3wz/vO5x)

[Radina, F. (2006) ‘Le sepolture 2 e 3 di Balsignano (Modugno, Bari) e la sepoltura 1 di masseria Maselli (Lama Balice, Bari)’, *La cultura del morire nelle societapreistoriche e protostoriche italiane. Studio interdisciplinare dei dati e loro trattamento informatico. Origines*, 1.](http://paperpile.com/b/QAa3wz/yVwL)

[Radina, F. *et al.* (2020) ‘Società neolitiche del sud-est italiano tra VI e V millennio aC: simboli e modelli di circolazione mediterranea nella documentazione funeraria’, *Società neolitiche del sud-est italiano tra VI e V millennio aC: simboli e modelli di circolazione mediterranea nella documentazione funeraria*, pp. 109–124.](http://paperpile.com/b/QAa3wz/1J39t)

[Robb, J. (2007) ‘Burial treatment as transformations of bodily ideology’, *Performing burial*, pp. 287–298.](http://paperpile.com/b/QAa3wz/2Mfed)

[Robb, J. and Mallegni, F. (1994) ‘Anthropology and paleopathology of Neolithic human remains from Catignano (Pescara, Italy)’, *Rivista di Antropologia*, 72, pp. 197–224.](http://paperpile.com/b/QAa3wz/pvfZh)

[Scarsini, C. *et al.* (2008) ‘Lo scheletro neolitico di Favella della Corte (Piana di Sibari, Cosenza)’, *Archivio per l’antropologia e la etnologia*, 138, pp. 47–69.](http://paperpile.com/b/QAa3wz/pfGZ)

[Scattarella, V. *et al.* (2002) ‘The individual of the early Neolithic of Balsignano (Bari, Italy): a study of some skeletal indicators of stress and palaeonutritional analysis’, *Human evolution*, 17(3), pp. 143–155.](http://paperpile.com/b/QAa3wz/tGASd)

[Sublimi Saponetti, S. *et al.* (2001) ‘Il villaggio neolitico trincerato di Malerba (Altamura): studio antropologico della tomba 2 e osservazione sui resti faunistici’, *‘Altamura’, Rivista storica, bollettino dell’Archivio-Biblioteca-Museo Civico, 42*, ‘Altamura’(42), pp. 9–40.](http://paperpile.com/b/QAa3wz/jq6TJ)

[Tafuri, M.A. *et al.* (2014) ‘Herding Practices in the Ditched Villages of the Neolithic Tavoliere (Apulia, South-east Italy): A Vicious Circle? The Isotopic Evidence’, in A. Whittle and P. Bickle (eds) *Early farmers*. British Academy, pp. 143–158.](http://paperpile.com/b/QAa3wz/SYZ0)

[Tinè, S. and Tinè, V. (1991) ‘Strutture di abitazione nel Neolitico Antico dell’Italia meridionale: la nuova evidenza da Favella di Sibari (CS)’. *13° Convegno Nazionale sulla Preistoria - Protostoria - Storia della Daunia*.](http://paperpile.com/b/QAa3wz/gkbOu)

[Tozzi, C. (2015) ‘Ripa Tetta. Lucera’, in A.M. Tunzi (ed.) *Venti del Neolitico Uomini del Rame. Preistoria della Puglia settentrionale.*, pp. 233–238.](http://paperpile.com/b/QAa3wz/Rdth)

[Tunzi, A.M. (1999) ‘La diga di Occhito’, in A.M. Tunzi (ed.) *Ipogei della Daunia. Preistoria di un territorio*. Claudio Grenzi Editore.](http://paperpile.com/b/QAa3wz/zUrR)

[Tunzi Sisto, A.M. and Sanseverino, R. (2007) ‘Insediamento neolitico in località La Torretta (Poggio Imperiale-FG)’, *Atti*, 28, pp. 71–86.](http://paperpile.com/b/QAa3wz/prTc)

[Tuross, N. (2012) ‘Comparative Decalcification Methods, Radiocarbon Dates, and Stable Isotopes of the VIRI Bones’, *Radiocarbon*, 54(3-4), pp. 837–844.](http://paperpile.com/b/QAa3wz/W7Iw)
