# Supplementary figures and images for: Regional long-term analysis of dietary isotopes in Neolithic southeastern Italy: new patterns and research directions
Source: Sci Rep. 2023 May 16;13:7914. doi: 10.1038/s41598-023-34771-y (PMC10188610; doi:10.1038/s41598-023-34771-y)

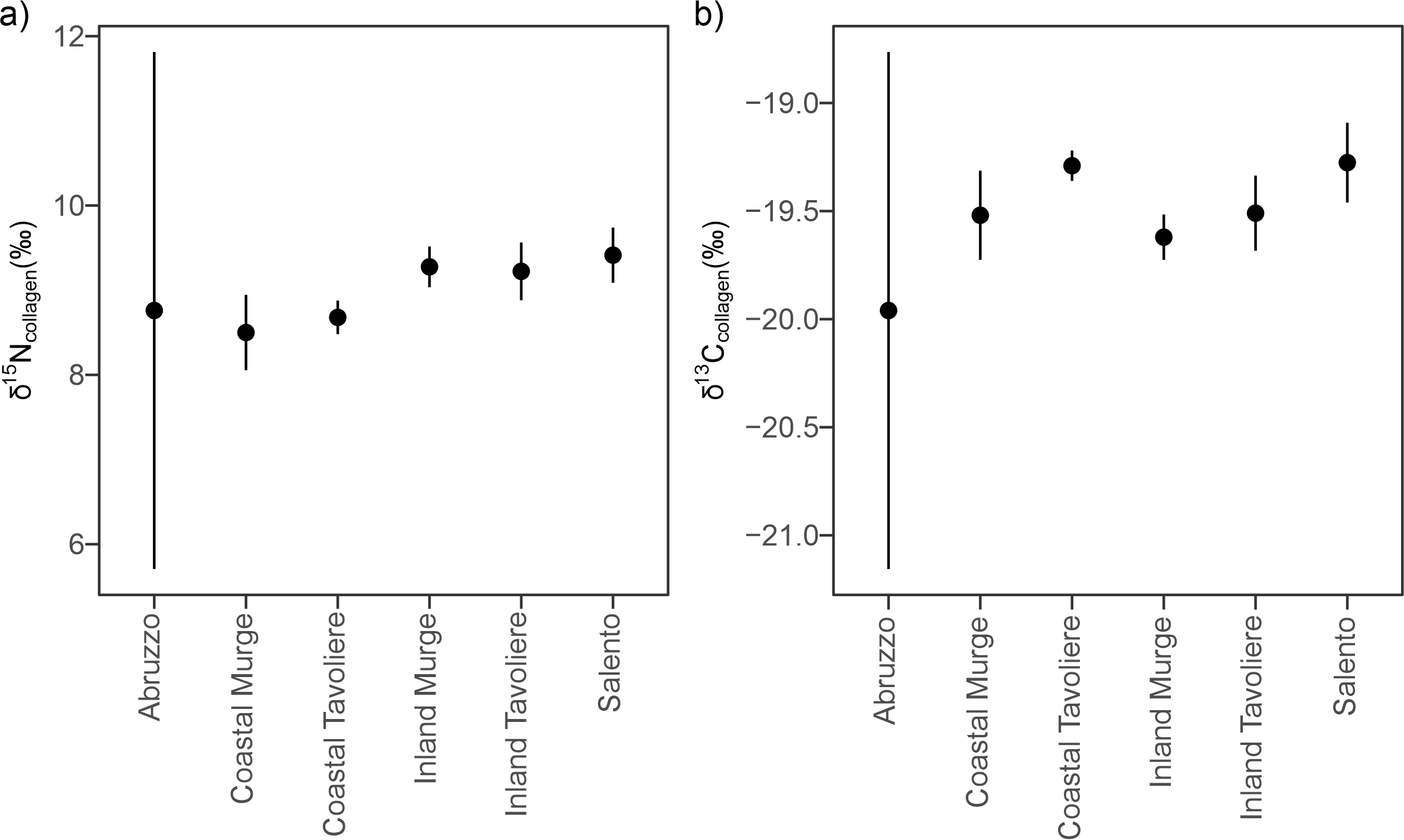

Supplement: Supplementary file 2 — Supplementary Information 1. [file 41598_2023_34771_MOESM2_ESM.png]

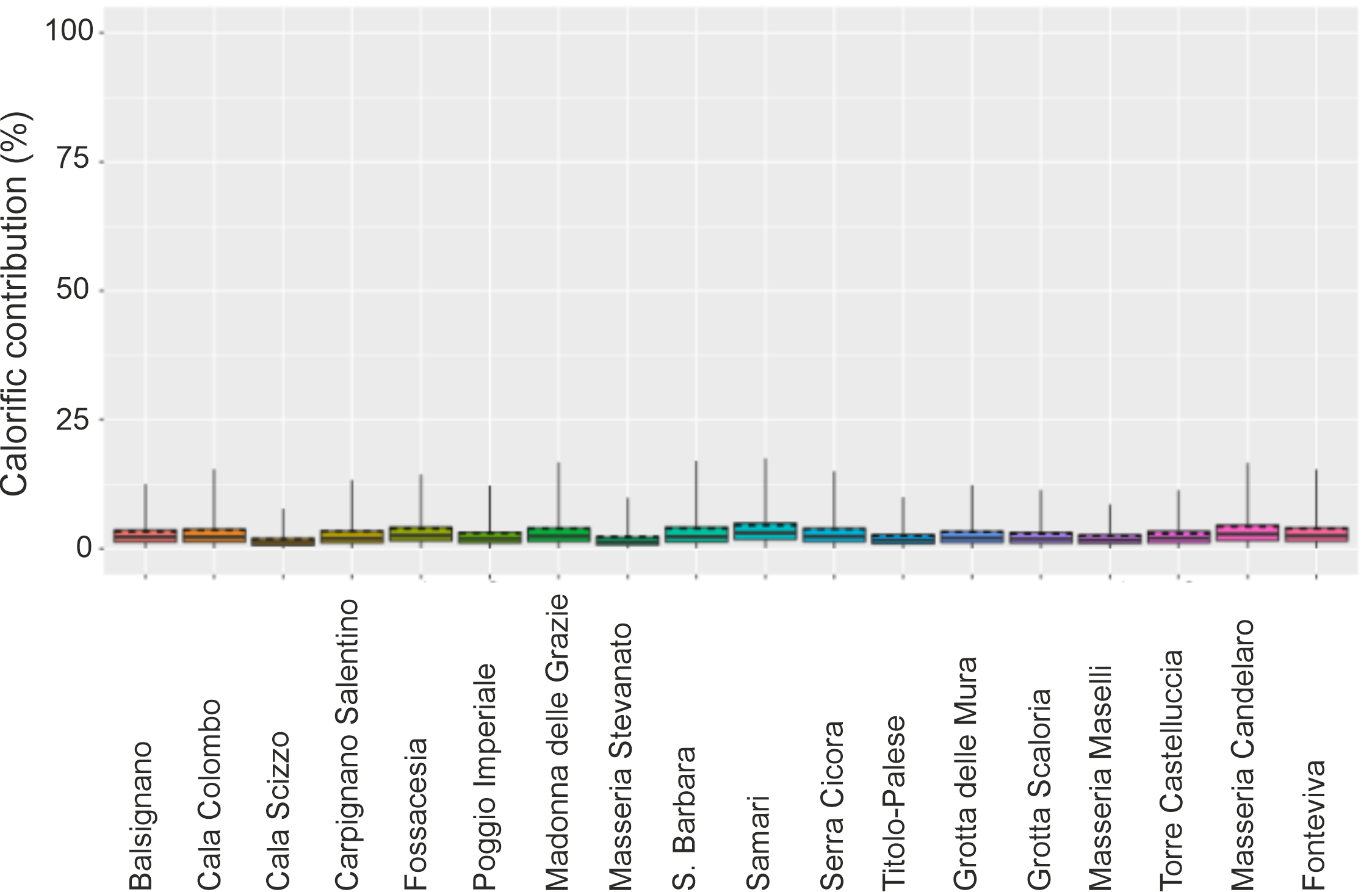

Supplement: Supplementary file 3 — Supplementary Information 2. [file 41598_2023_34771_MOESM3_ESM.png]

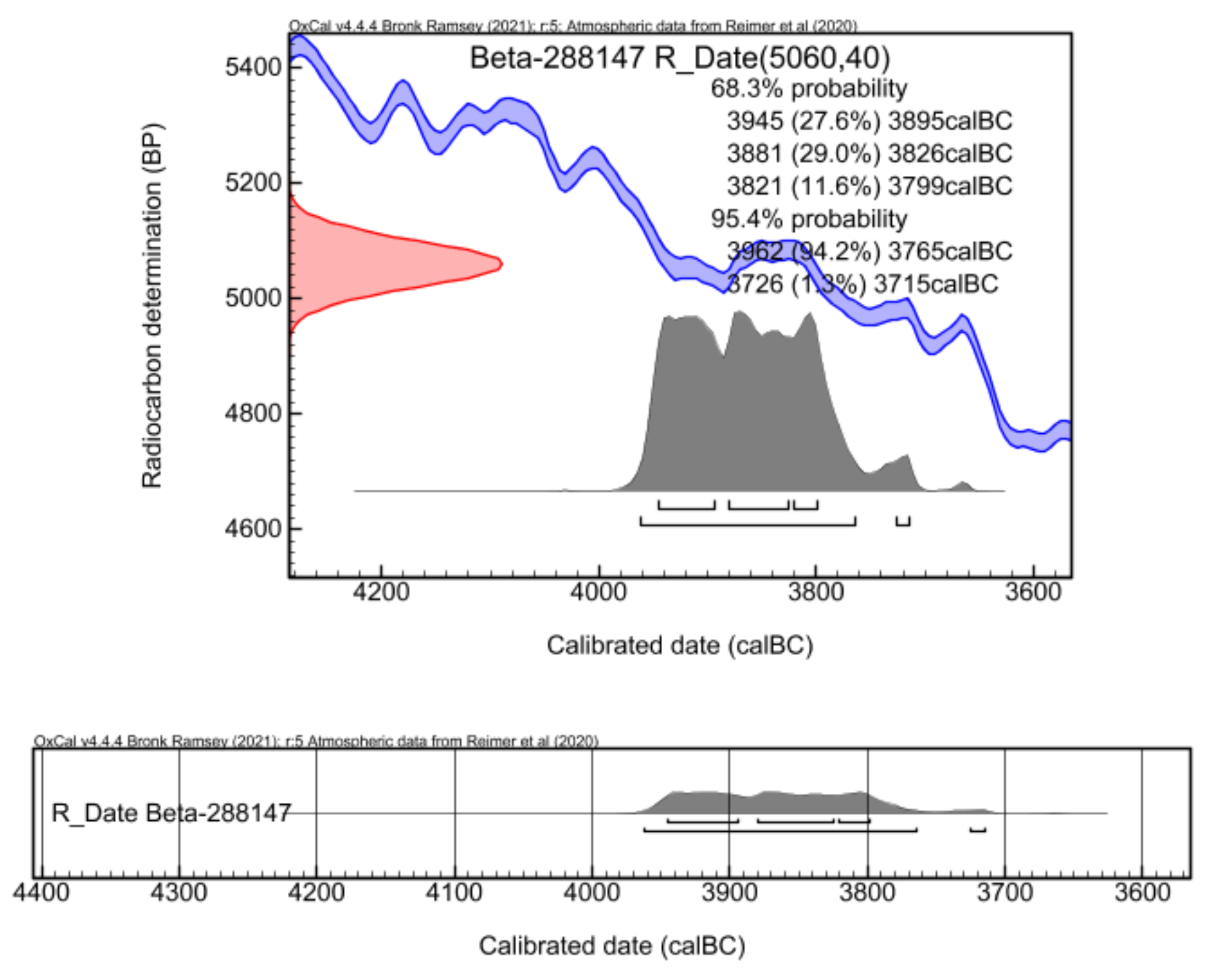

Supplement: Supplementary file 4 — Supplementary Information 3. [file 41598_2023_34771_MOESM4_ESM.png]
