# Supplementary material for: Regional long-term analysis of dietary isotopes in Neolithic southeastern Italy: new patterns and research directions
Source: Sci Rep. 2023 May 16;13:7914. doi: 10.1038/s41598-023-34771-y (PMC10188610; doi:10.1038/s41598-023-34771-y)
Supplement: Supplementary file 10 — Supplementary Information 9. [file 41598_2023_34771_MOESM10_ESM.zip › help.html]

**Welcome to the FRUITS App!**

This is the help
